# Metabolomics and Machine Learning Approaches Combined in Pursuit for More Accurate Paracoccidioidomycosis Diagnoses

**DOI:** 10.1128/mSystems.00258-20

**Published:** 2020-06-30

**Authors:** Estela de Oliveira Lima, Luiz Claudio Navarro, Karen Noda Morishita, Camila Mika Kamikawa, Rafael Gustavo Martins Rodrigues, Mohamed Ziad Dabaja, Diogo Noin de Oliveira, Jeany Delafiori, Flávia Luísa Dias-Audibert, Marta da Silva Ribeiro, Adriana Pardini Vicentini, Anderson Rocha, Rodrigo Ramos Catharino

**Affiliations:** a Department of Internal Medicine, Botucatu Medical School, São Paulo State University, Botucatu, SP, Brazil; b Innovare Biomarkers Laboratory, School of Pharmaceutical Sciences, University of Campinas, Campinas, SP, Brazil; c RECOD Laboratory, Institute of Computing, University of Campinas, Campinas, SP, Brazil; d Laboratory of Mycosis Immunodiagnosis—Immunology Section, Adolfo Lutz Institute, São Paulo, SP, Brazil; University of California, San Diego

**Keywords:** artificial intelligence, diagnosis, metabolomics, paracoccidioidomycosis

## Abstract

Brazil and many other Latin American countries are areas of endemicity for different neglected diseases, and the fungal infection paracoccidioidomycosis (PCM) is one of them. Among the clinical manifestations, pneumopathy associated with skin and mucosal lesions is the most frequent. PCM definitive diagnosis depends on yeast microscopic visualization and immunological tests, but both present ambiguous results and difficulty in differentiating PCM from other fungal infections. This research has employed metabolomics analysis through high-resolution mass spectrometry to identify PCM biomarkers in serum samples in order to improve diagnosis for this debilitating disease. To upgrade the biomarker selection, machine learning approaches, using Random Forest classifiers, were combined with metabolomics data analysis. The proposed combination of these two analytical methods resulted in the identification of a set of 19 PCM biomarkers that show accuracy of 97.1%, specificity of 100%, and sensitivity of 94.1%. The obtained results are promising and present great potential to improve PCM definitive diagnosis and adequate pharmacological treatment, reducing the incidence of PCM sequelae and resulting in a better quality of life.

**IMPORTANCE** Paracoccidioidomycosis (PCM) is a fungal infection typically found in Latin American countries, especially in Brazil. The identification of this disease is based on techniques that may fail sometimes. Intending to improve PCM detection in patient samples, this study used the combination of two of the newest technologies, artificial intelligence and metabolomics. This combination allowed PCM detection, independently of disease form, through identification of a set of molecules present in patients’ blood. The great difference in this research was the ability to detect disease with better confidence than the routine methods employed today. Another important point is that among the molecules, it was possible to identify some indicators of contamination and other infection that might worsen patients’ condition. Thus, the present work shows a great potential to improve PCM diagnosis and even disease management, considering the possibility to identify concomitant harmful factors.

## Abstract (precis)

Paracoccidioidomycosis (PCM) is a fungal infection typically found in Latin American countries, especially in Brazil. The identification of this disease is based on techniques that may fail sometimes. Intending to improve PCM detection in patient samples, this study used the combination of two of the newest technologies, artificial intelligence and metabolomics. This combination allowed PCM detection, independently of disease form, through identification of a set of molecules present in patients’ blood. The great difference in this research was the ability to detect disease with better confidence than the routine methods employed today. Another important point is that among the molecules, it was possible to identify some indicators of contamination and other infection that might worsen patients’ condition. Thus, the present work shows a great potential to improve PCM diagnosis and even disease management, considering the possibility to identify concomitant harmful factors.

## INTRODUCTION

Pathogenic fungi have been a matter of concern worldwide, but in Latin America, a particular soilborne and thermally dimorphic fungus, Paracoccidioides brasiliensis, is responsible for the most frequent systemic mycoses (1, 2). It is most prevalent in Brazil, where 80% of paracoccidioidomycosis (PCM) cases are reported (3, 4). Another species has been recently identified within the *Paracoccidioides* genus, Paracoccidioides lutzii, which has been associated with some paracoccidioidomycosis (PCM) cases. This fungal disease is acquired by inhalation of fungal conidia through the respiratory tract, where it is converted to its yeast form and can be arrested by innate immunity cells, forming granulomas, or can spread via the bloodstream and lymphatic system and progress to systemic granulomatous disease with mucocutaneous lesions and visceral injuries (5).

PCM can manifest in two clinical forms, acute/subacute and chronic forms. The first one affects mainly children, teenagers, and young adults; in this case, fungal infection targets mainly the mononuclear phagocytic system, and lymph nodes, spleen, bone marrow, and liver are the most affected organs. However, the PCM chronic form affects adults of 30 years or more and takes from months to years to show signs or symptoms, and patients might present pulmonary insufficiency, malnutrition, and disseminated lesions, affecting skin and oral mucosa as well. In this context of diverse manifestations, the definitive diagnosis impacts directly strategies for PCM treatment, reduction of frequent sequelae, and better quality of life (6, 7).

Conventional diagnosis of PCM is based on the presence of *Paracoccidioides* spp. in clinical samples, called mycological diagnosis. It consists of the visualization of fungal morphological structures through optical microscopy by either tissue or sputum analysis (8). The sensitivity of sputum mycological evaluation ranges from 63% to 95%, according to the sample preparation method; the sensitivity of histopathological evaluation may achieve 97%. Despite these sensitivity indexes, specificity remains an Achilles’ heel for mycological diagnosis, since *Paracoccidioides* morphology resembles other species, especially Histoplasma capsulatum and Cryptococcus neoformans, hampering the correct identification and diagnosis through microscopic evaluation (9).

In addition to mycological diagnosis, serological tests have been developed and applied. Serological tests are based on antibody detection from patients’ serum; however, there is an essential issue with accuracy, because cross-reactivity is continually observed, mainly with infections such as histoplasmosis, cryptococcosis, and aspergillosis (5). The growing development of molecular biology has brought new molecular tests for PCM diagnosis, which are mainly based on PCR and nested PCR. These molecular techniques are still searching for better targets that allow standardization and application with suitable accuracy parameters; besides, they are not available for routine diagnosis (10). On the other hand, metabolomics has emerged as an innovative analytical instrument, presenting high sensitivity to detect a broad and diverse range of molecules that represent the phenotype of living organisms, for either health or illness contexts (11).

The present research proposes a new method based on the metabolomics approach to improve PCM diagnostic accuracy and reduce the cost-benefit ratio, regardless of the PCM form. Although untargeted metabolomics is capable of revealing the slighter alterations in metabolite profile of a specific disease, it provides a considerable amount of metabolic information, which depends on robust methods for analysis and classification of features (12). Data mining techniques, which include machine learning, statistical analysis, and database management systems, can extract implicit patterns from massive biological data sets and, in the last decade, have been applied to identify characteristic molecular features for each condition (13, 14).

Therefore, metabolomics techniques were combined with a machine learning prediction model aimed at extracting from biological data a typical and consistent molecular pattern for PCM. For that, the present work performed a metabolomics approach through high-resolution mass spectrometry (HRMS) analysis attached to a machine learning classification algorithm. To identify the most critical metabolic features, mass spectrometry big data were input for training a decision-making algorithm. The combination of biochemical analysis and artificial intelligence allowed us to observe a set of features that depict PCM condition, independent of disease stage.

## RESULTS

### Selection of potential biomarkers through machine learning.

The machine learning method for biomarker determination described in Materials and Methods was applied over the spectrum data as follows.

The collection of 1,708 spectrum vectors of *m/z* intensities, resulting from the spectrometry quintuplicate measurements of biological samples of 343 individuals, was normalized dividing each intensity by the highest absolute intensity on the vector (normalization where maximum equals 1), and patients’ samples were randomly split into fit partition (Pfit) and test partition (Ptest) in the proportion of 80% and 20%, respectively. Classifiers were trained and validated in all steps of the method using 10 experiments of Pfit randomly shuffled and divided into training partition (Ptrain) and validation partition (Pval) in the proportions of 80% and 20%, respectively.

**FIG 1:**
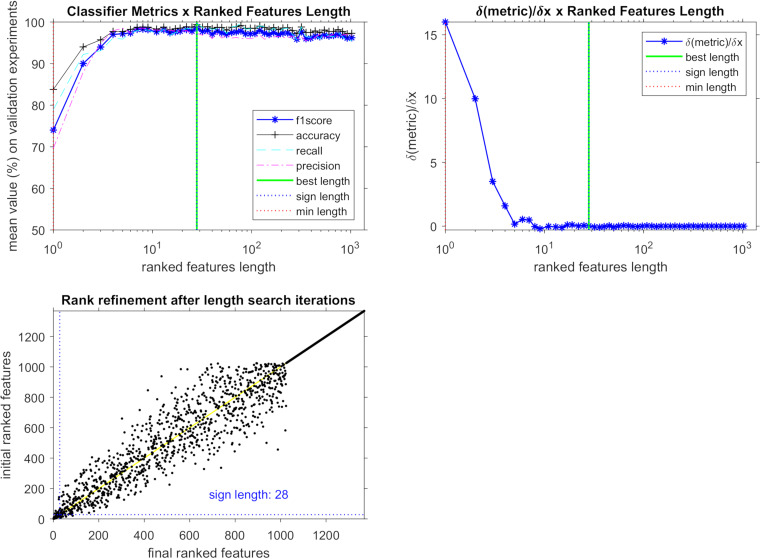
Optimization process to determine the most important features. The sign length indicates the number of variables which are the most discriminant ones. (Top left) Results of the iteration process as a function of the ranked feature length, determining 28 features for the best F1 score achieved. (Top right) Approximate derivative of the F1 score curve showing that results start becoming almost stable above the 10 first-ranked features. (Bottom left) Initial ranked features versus final ranked features, demonstrating the rank refinement convergence and that most discriminant features are in the first positions from the beginning of the process.

**FIG 2:**
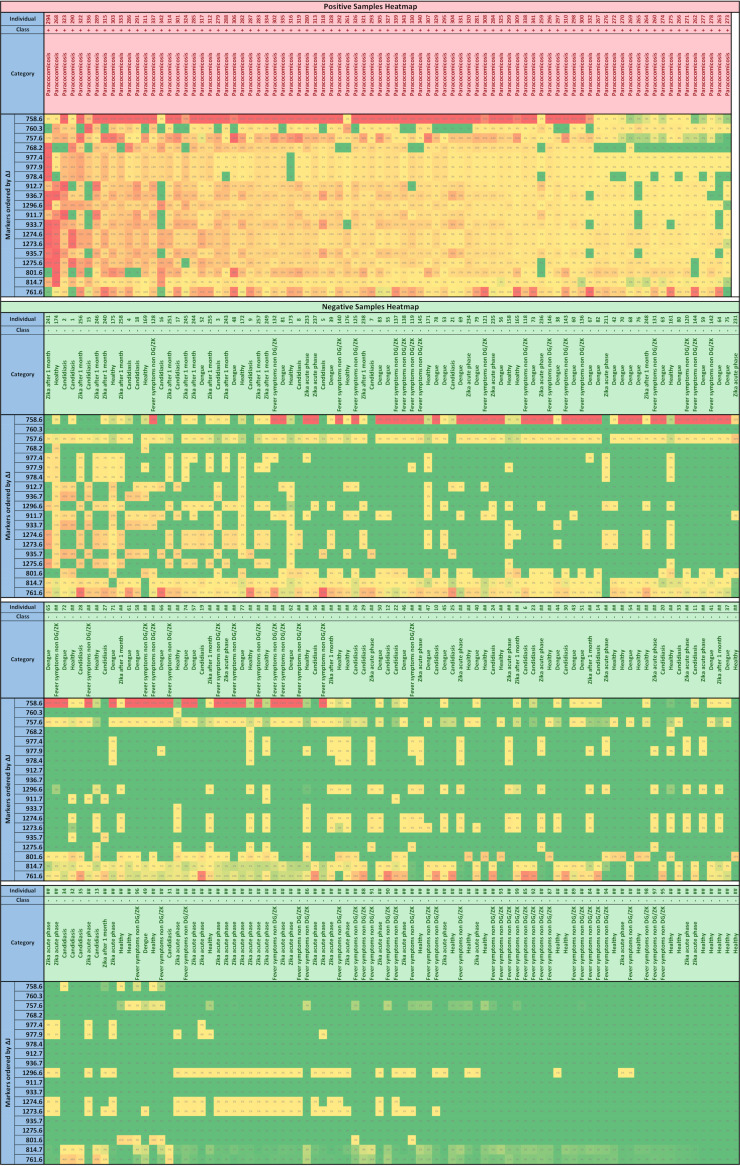
Heatmap of the 19 most discriminant features. The color scale is from dark green (0%) to dark red (100%), corresponding to the minimum and maximum intensity values of the marker *m/z* on all samples, respectively.

Figure 1 depicts the evolution of metrics as the vector shrinks by discarding the less important features. Statistical metric definitions are shown in Table 1. The best results were achieved with the length of 28 features (Table 2). Table 3 shows the metrics for the most-discriminant feature point and also for the marker-selected ones. Even though 28 features were identified by the classifier as responsible for maximizing the prediction result, some of them were not considered actual PCM markers (Table 2) by the Δ*J* criterion, by which a marker should have a higher probability to present higher intensities on the PCM-infected patients. Using the Δ*J* criterion, 19 PCM candidate biomarkers were selected. Although the highest values of accuracy, sensitivity, and specificity were achieved during validation testing with 28 best-length features (Table 3), there was no statistically significant difference for the same metrics when only the 19 PCM candidate biomarkers were evaluated in the final test. In this way, we focused on elucidation of these 19 features intending to understand the PCM pathophysiology and looking for a specific yeast biomarker. In Fig. 2, a heatmap shows the 19 most significant *m/z* features associated with PCM condition (according to Δ*J* rank [Table 3]) and their relevance for other individuals’ health conditions.

**TABLE 1:** Definitions of statistical metrics to evaluate classification results^*a*^

Metric	Abbreviation	Formula
Sensitivity	STV or TPR	TP/(TP + FN)
Specificity	SPC	TN/(TN + FP)
Precision	PRC	TP/(TP + FP)
F1 score	F1S	2 PRC STV/(PRC + STV)
Accuracy	ACC	(STV + SPC)/2

a Abbreviations: TP, true positives; TN, true negatives; FP, false positives; FN, false negatives.

**TABLE 2:** The 28 most discriminant features which, together, achieved the best prediction performance

Rank	Marker	*m/z*	Δ*J* (%)
1	Yes	1,274.6	47.0
2	Yes	912.7	48.1
3	Yes	760.3	50.0
4	No	808.5	0.0
5	Yes	977.9	49.1
6	Yes	1,275.6	46.1
7	No	909.8	0.0
8	Yes	935.7	46.1
9	Yes	814.7	41.2
10	No	822.4	0.0
11	Yes	1,273.6	46.7
12	Yes	977.4	49.1
13	Yes	758.6	50.0
14	Yes	936.7	47.8
15	No	1,276.6	0.0
16	Yes	801.6	45.6
17	Yes	911.7	47.5
18	Yes	757.6	49.5
19	Yes	1,296.6	47.7
20	No	860.3	0.0
21	Yes	761.6	33.9
22	No	839.6	24.8
23	Yes	768.2	49.4
24	No	1,046.4	0.0
25	No	1,045.4	0.0
26	No	760.5	0.0
27	Yes	978.4	49.1
28	Yes	933.7	47.3

**TABLE 3:** Classification results of the validation tests and the final test using the 28 most discriminant features and the 19 PCM biomarkers according to Δ*J* rank

Metric	Signature (best length)			Only markers		
	Validation		Final test	Validation		Final test
	Mean	SD		Mean	SD	
Vector length	28		28	19		19
No. of trees	76		76	73		73
Accuracy (%)	99.0	1.0	97.1	97.4	2.3	97.1
Sensitivity (%)	99.3	2.1	94.1	96.3	4.9	94.1
Specificity (%)	98.8	1.3	100.0	98.6	1.3	100.0
Precision (%)	96.4	3.7	100.0	95.6	3.8	100.0
F1 score (%)	97.8	1.9	97.0	96.0	2.7	97.0

### Metabolomics and identification of biomarkers.

From *m/z* values, metabolite analyses were performed using metabolomics databases and literature search to elucidate the selected features and better understand metabolism upon *P. brasiliensis* infection. Table 4 presents 15 putative markers, proposed from the set of 19 PCM features; this reduction in the number of candidate markers is expected since the full set of 19 PCM features encompassed ions that were relative to the isotopes of a single molecule. In this study, we could observe 4 isotopes among the selected biomarkers. For example, ions 1273.6, 1274.6, and 1275.6 are relative to the isotopic distribution of a single molecule, *m/z* 1273.6, as confirmed by the associated spectra upon visual inspection. Thus, the list of candidate markers includes a mycotoxin (*m/z* 760.3), glycosphingolipids (GSLs) (*m/z* 756.6 [most abundant ion corresponding to isotope 757.6] and *m/z* 758.6), phosphosphingolipid (*m/z* 761.6), triacylglycerols (*m/z* 911.7 [and respective isotope 912.7], 933.7, 935.7 [and respective isotope 936.7], 801.6, and 814.7), and prenyl lipids (*m/z* 1273.6 [and respective isotopes 1274.6 and 1275.6]). Five other potential metabolites were also selected as PCM biomarkers: *m/z* 768.2, 977.4, 977.9, 978.4, and 1296.6. However, we were not able to elucidate these species using the available tools. Considering the fact that this work is nontargeted metabolomics, the lack of database matches for some of the selected markers is not uncommon (11). Therefore, these features were classified as unknown biomarkers, albeit they remain as important metabolites in PCM detection for the machine learning method.

**TABLE 4:** Molecular features selected by machine learning analysis in PCM patients’ serum samples^*b*^

Molecule	ID	Theoretical mass (Da)	Exptl mass (Da)	Adduct	Error (ppm)	MS/MS (*m*/*z*)
Fumonisin B1 and/or isofumonisin B1	MID 53922, MID 88649	760.3516	760.3506	[M + K]^+^	1.31	508, 714, 506, 572
Cerebroside D	MID 477	756.5984	756.5997	[M + H]^+^	−1.71	710, 738, 568
GlcCer (d36:1^*a*^ [2OH])	LMSP05010059	758.6141	758.6151	[M + H]^+^	−1.31	686, 570, 519
PE-Cer (40:1^*a*^ [2OH])	MID 103125	761.6167	761.6152	[M + H]^+^	1.96	508, 536, 729
TG (53:2)^*a*^	MID 36808	911.7464	911.7482	[M + K]^+^	−1.97	655, 629, 335, 865
TG (55:4)^*a*^	MID 100332	935.7464	935.7478	[M + K]^+^	−1.49	653, 679, 639, 903
TG (55:5)^*a*^	MID 101029	933.7308	933.7317	[M + K]^+^	−0.96	651, 677, 637, 683
TG (48:3)^*a*^	MID 99740	801.6967	801.6981	[M + H]^+^	−1.74	551, 729, 545, 567
TG (49:4)^*a*^	MID 100470	813.6967	813.6983	[M + H]^+^	−1.96	795, 557, 781, 681
Rhamnosyl-galactosyl-diphosphoundecaprenol	MID 71958	1,273.7057	1,273.7033	[M + K]^+^	1,88	1,258, 1,253, 1,231
Unknown			768.2646			
Unknown			977.4555			
Unknown			977.9566			
Unknown			978.4537			
Unknown			1,296.6092			

a Carbon number: double bond.

b Abbreviations: M, molecule mass without adduct; ppm, parts per million; MS/MS, tandem mass spectrometry; MID, Metlin ID; LMSP, Lipid Maps data bank ID; TG, triacylglyceride; GlcCer, glucosylceramide; PE-Cer, phosphoethanolamine-ceramide.

After metabolomics analysis, three biomarkers were selected as the most compatible ones with the metabolites of Paracoccidioides brasiliensis. Therefore, we have performed an oriented classifier training with 756.6 and 758.6 (Table 5), and only these two biomarkers were able to predict paracoccidioidomycosis with most of the metrics above 85%.

**TABLE 5:** Classification results of the validation tests and the final test using the features 756.6 and 758.6 as the most representative of PCM condition

Metric	756.6, 758.6		
	Validation		Final test
	Mean	SD	
Vector length	2		2
No. of trees	64		64
Accuracy (%)	86.2	6.9	92.2
Sensitivity (%)	79.4	14.4	88.2
Specificity (%)	93.0	3.3	96.1
Precision (%)	78.8	7.4	88.2
F1 score (%)	79.1	8.5	88.2

## DISCUSSION

The present research allowed use of a machine learning method to select potential PCM biomarkers aimed at achieving better accuracy, sensitivity, and specificity metrics than the routine available methods. This approach enabled the identification of diverse biomarkers which are discussed below.

Among them, a mycotoxin was selected in PCM patients’ serum samples that was known as fumonisin, a toxin typically produced by *Fusarium* fungal species, which are frequently found in maize kernels (15). Economically, fumonisin B1 is considered the most harmful mycotoxin among fumonisins in Brazil, the third largest maize producer worldwide (16). Due to climatic characteristics, especially in Brazil’s central and southern regions, *Fusarium* spp. are commonly found in maize fields, or even after harvest or during storage (17, 18). Interestingly, the areas of prevalence of PCM cases are coincident with the main agricultural area for grains, especially maize (5); therefore, infection by *P. brasiliensis* concomitant with fumonisin contamination has been proposed (19, 20). Besides, fumonisins are known as modulators of mammals’ immune responses, downregulating phagocytic activity and increasing antibody specificity against Paracoccidioides brasiliensis (20, 21). Consequently, the cooccurrence of PCM and fumonisin contamination might negatively influence cellular immune response and worsen patients’ clinical manifestations.

Among the identified PCM metabolites, cerebroside D and a glucosylceramide (GlcCer) were selected as important glycosphingolipids (GSLs) in patients’ serum. Different studies have shown that glucosylceramide backbones, present in these biomarkers, are involved in host-pathogen interaction and may be associated with *P. brasiliensis* antigenicity (22). Some characteristics observed in both the sphingolipids cerebroside D and GlcCer are typical in glucosylceramide from fungi, such as a methyl linked to the sphingosine chain and an (E)-Δ8-unsaturation. Cerebroside D presents another fungal characteristic which is the presence of another Δ4-unsaturation in the ceramide moiety (23, 24). In addition, cerebrosides are neutral glycosphingolipids widely found in pathogenic fungi and are involved in many cellular processes, as well as signaling, differentiation, growth (25), and antigenic activity (23, 26). Different immunological and analytical methods have also evaluated Paracoccidioides brasiliensis glucosylceramides and observed that cerebroside D was identified not only in mycelium but also in yeast samples, the pathogenic form (23, 27). Therefore, these data corroborate our findings and indicate that cerebroside D and GlcCer are *P. brasiliensis* biomarkers present in patients’ serum samples.

Still in the sphingolipid class, phosphoethanolamine-ceramide (PE-Cer) was also selected as an important marker for detection of paracoccidioidomycosis. PE-Cer is a byproduct of sphingolipid metabolism through the sphingomyelinase pathway, where sphingomyelin, hydrolyzed into *N*-acylsphingosine (ceramide), is a precursor of PE-Cer (28, 29). Sphingomyelin represents one of the most abundant sphingolipids that assemble the cellular membrane (30, 31). Once its metabolites, especially ceramide derivates, are significantly present in biological samples, that indicates that cellular membranes are seriously damaged. Some studies have shown that conversion of sphingomyelin into ceramide and its derivatives is associated with apoptosis (32, 33), especially C_16_ ceramide, which corresponds to our selected PE-Cer (34). Besides, healthy lungs present lower ceramide levels than lungs with chronic obstructive pulmonary disease (35), which corroborates our findings that paracoccidioidomycosis affects sphingolipid metabolism and increases ceramide derivatives which, through cell death, reduce lung function and induce pulmonary manifestations.

Apart from sphingolipids, polyprenyl lipids and phosphorylated derivatives represent a small portion of glycerophospholipids in cellular membranes mainly found in bacteria, fungi, and plants. The selected phosphorylated polyprenyl lipid (*m/z* 1273.7) corresponds to a lipid carrier, undecaprenol, which is a 55-carbon-chain isoprenol (C_55_-P) used by prokaryotes in sugar carrier processes to build polysaccharide structures such as, for example, peptidoglycan (36). PCM patients may present genetic disorders that predispose to coinfections, for example, infections caused by mycobacteria, known as Mendelian susceptibility for mycobacterial diseases (MSMD) (5, 37). Facing some genetic polymorphisms, PCM patients may present decreased levels of some cytokines, such as interferon gamma (IFN-γ) (38, 39). Taking that into account, it is expected that the individual’s immune system might be impaired and therefore be susceptible to coinfections. Therefore, it is plausible to find bacterial biomarkers in PCM patients, as coinfection is a possible condition in PCM since IFN-γ has an essential role in resistance to bacteria and resolution of infections as a macrophage activator (40, 41). Although C_55_-PP is typically found in bacteria, polyprenyl-phosphate lipids are involved in protein glycosylation in all kingdoms of life, even eukaryotes (36, 42). Further studies are necessary to evaluate the possibility of considering undecaprenol as a glycan lipid carrier in yeasts, which may be associated with glycosylation of Paracoccidioides brasiliensis’ main antigen, a 43,000-Da glycoprotein (GP43).

Last, but not least, triacylglycerides were also considered relevant biomarkers in our study. Paracoccidioides brasiliensis is known to reprogram its metabolic pathways to improve the energetic supply. During mouse lung infection, the yeast cells showed upregulation of enzymes involved in lipid oxidation. For example, acetyl coenzyme A (acetyl-CoA) and propionyl-CoA (both derived from lipid catabolism) are used during *P. brasiliensis* infection to fuel the glyoxylate cycle and provide a supply for synthesis of biomolecules (43). Besides, proteomic studies have also shown that yeast cells, once internalized by macrophages, present fatty acid degradation and its usage as fuel for survival inside phagocytic cells (44). Therefore, PCM might increase the need for systemic triacylglycerides, probably induced by the energetic yeast demand.

Analyzing all the results, it was possible to identify a set of 28 features from which the applied method could select 19 PCM biomarkers, among them 13 molecules and their respective isotopes. Together, these markers are reliable indicators of PCM with 100% specificity, 94.1% sensitivity, and 97.1% accuracy. The 19 PCM markers were then elucidated according to metabolomics analysis. Next, it was observed that, among the 19 features, three biochemical markers were the most significant ones in our screening, and their specificity and accuracy were greater than 95%. These data show that, independently of fungal disease form and according to the predetermined set of discriminant biomarkers, it is possible to reach metrics such as 100% specificity, 94.1% sensitivity, and 97.1% accuracy, higher indexes than traditional microbiological and serological methods. In addition, some of the 19 features may be essential indicators of cooccurrence of infection or contamination, which opens a new alternative for applying metabolomics analysis to improve diagnosis and therapeutic approaches and ensure treatment confidence.

The present work consists of a biomarker screening test for PCM diagnosis through the association of different techniques: machine learning and mass spectrometry. The next step for further test refinement is the inclusion of more patients, especially with different systemic mycoses and lung infections (i.e., tuberculosis) to strengthen the validation for laboratory diagnosis. Microbiologists and other health professionals will be able to use this method easily and cheaply. We intend to make it possible through a software solution that will be combined with mass spectrometers which, together, will predict samples positive or negative for PCM. Therefore, we propose to complete the sample set, increasing diversity and quantity and intending to magnify test confidence; retrain the mathematical model; and validate it for further application in clinical laboratories. In this way, this neglected disease might have a chance to be rapidly identified and recognized, enabling patients to receive proper medical care and reducing the sequelae and its social impact, mainly in individual work capacity and quality of life.

## MATERIALS AND METHODS

### Ethics statement.

This work was approved under number 1.850.251 by the Research Ethics Committee of the University of Campinas. The patients were informed about the study through an approved consent form, and this study was conducted according to the principles expressed in the Declaration of Helsinki.

### Research participants and specimen collection.

In total, 343 individuals were included in this study, regardless of age and gender, in two main groups: the test group, consisting of PCM patients (*n* = 85), and the control group (*n* = 258). Aiming to increase the diversity in the control group, the latter was formed of healthy volunteers (*n* = 47) and patients with different infectious diseases—candidemia (*n* = 36), dengue (*n* = 47), Zika virus (ZIKV) infection (*n* = 65), and finally a group of people with fever symptoms but not dengue or Zika virus infection (*n* = 63)—comprising a total of 258 samples, all negative for Paracoccidioides brasiliensis. The PCM group was established according to previous patient’s serological tests with a positive reaction, done by the Adolfo Lutz Institute-Laboratory of Mycosis Immunodiagnosis, independently of paracoccidioidomycosis form. All the other infections were diagnosed according to the gold standard methods recommended for each one, including real-time PCR and microbiological culture tests.

### PCM detection by AGID.

Paracoccidioidomycosis was previously detected by a serological test based on agar gel immunodiffusion (AGID), according to the method of Kamikawa (45).

### HRMS preparation and analysis.

Starting from 20 μl of serum samples, prepared according to the method of Melo et al. (46, 47), patients and control groups were evaluated in quintuplicates through direct injection into a high-resolution mass spectrometer (HRMS; ESI-LTQ-XL Orbitrap Discovery instrument [Thermo Scientific, Bremen, Germany]). Instrumentation parameters were set as follows: sample flow of 10 μl/min, sheath gas at 10 arbitrary units, source voltage of 5 kV, and capillary temperature of 280°C. Analytical quintuplicates were prepared and analyzed for each sample, from which metabolic fingerprints were captured in the mass range of 750 to 1,700 *m/z* in the positive ion mode.

Intending to confirm our findings, tandem mass spectrometry (MS/MS) was applied in the same instrument mentioned above. The collision gas used was helium, with collision-induced dissociation energy ranging from 30 to 60 (arbitrary units). The obtained experimental mass fragmentation spectra were collected and compared to *in silico* mass fragmentation profiles of each marker, simulated with Mass Frontier software (v. 6.0; Thermo Scientific, San Jose, CA).

### Database search.

The selected metabolic features were elucidated through a search on METLIN (Scripps Center for Metabolomics, La Jolla, CA), on the Lipid Maps database, and in literature.

### Machine learning method.

Forests of decision trees are one of the best prediction algorithms in different areas of knowledge (48, 49). They were proposed by Breiman (50), who developed and trademarked them as Random Forests. The method consists of combining results of many trained decision trees (bagging strategy) (51) using a subset of the data space (bootstrap strategy). The data’s subspace is selected for training each decision tree through a random subset of the variables (dimensional subset) and a random subset of the data vectors (points subset). Each node in the decision trees tests one variable against a cutting decision value. The cutting value determines a plan in the hyperspace, which is orthogonal to the variable’s dimension and splits the space into two subspaces. The algorithm searches for the cutting values that increase the information gain on each decision node achieving a prediction value when a leaf is reached. A complete review of decision tree classifiers can be found in reference 52, and a probabilistic (Bayesian) explanation of them can be found in reference 53.

The Random Forest algorithm deals with multivariate nonlinear problems with simple parameterization. Its parameters can be adjusted to enhance the prediction performance and computation footprint, such as the number of trained trees in the forest, the size of the variable subspace used in each training, depth of each tree, and pruning strategy, among others (54).

With the classifier trained, the classification is a simple sequence of tests traversing the decisions in the forest and combining the results by majority voting (mode of the classification results) or by another aggregation method. With this fusion strategy, Random Forest classifiers yield a robust-to-noise performance in the prediction for new data.

Another advantage of using the Random Forest algorithm is the ability to identify which variables contribute more to the prediction results, i.e., what variables have more determinant impact in the forecast performance statistics (accuracy, precision, and others). This property, known as variable importance (or feature importance) (55, 56), is particularly essential on the metabolomics studies conducted in this paper, as the starting point for the metabolomics analysis is to discover which molecules represented in the spectrum data drive the successful predictions of the algorithm.

The machine learning approach used in this paper is similar to the method applied and already described in a paper on ZIKV detection (57), which successfully identified in the blood serum molecules associated with the virus metabolic process.

The analysis method consists of training a Random Forest classifier using labeled data: the already-diagnosed condition of PMC infection (positive samples) and noninfection (control, negative samples), refining the process and selecting *m/z* variables until the best prediction performance is achieved.

The variable selection process uses the Random Forest feature importance process to discard less discriminant features and consequently to identify the most important ones that drive the method to the best classification results. The optimization algorithm searches for the maximization of the cost metric (e.g., the F1 score measure [Table 1]) discarding, in each iteration, the 10% least discriminant ones. The feature importance calculation used in this process employs the variable permutation algorithm, which is the best way (56) to compute the contribution of each feature in the classification result.

As the most discriminant *m/z* values are determined, a statistical distribution analysis over the intensity of the corresponding ions determines which ones are more frequently present in the infected patients than the control ones, identifying the possible biomarkers for the disease. This is an essential step in the preparation for the metabolomics analysis, as it narrows down the possible biomarkers to a small number of molecules that makes the biochemical analysis and metabolic process determination feasible and faster.

Possible biomarkers are the most discriminant features (determined by the learning process) for which the intensity value cumulative distribution function (CDF) of the negative patients computed at the intensity value of the median of positive patients is over Δ*J*% of the CDF’s positive patients. It indicates that the probability of finding a higher intensity of the *m/z* in the spectra of positive patients is much higher than in the negative ones, which we consider the evidence of a possible biomarker, which will be validated by the subsequent metabolomics investigation.

*F_j_* is a marker feature, if

$$\Delta_{j}=\left(1-P\left(m_{j}\right)\right)-\left(1-Q\left(m_{j}\right)\right)=Q\left(m_{j}\right)-P\left(m_{j}\right)>\text{β}$$

and

$$Q\left(F_{j}\right)\geq P\left(F_{j}\right)|\forall F_{j}>m_{j}$$

where *y_j_* is an *F_j_* value for a positive patient, *m_j_* is the median of *F_j_* values of all positive patients, $\bar{y_{j}}$ is an *F_j_* value for a negative patient, *p*(*y_j_*) is the probability distribution function of positive patients, $q\left(\bar{y_{j}}\right)$ is the probability distribution function of negative patients, *P*(*y_j_*) is the cumulative distribution function (CDF) of *y* values, and $Q\left(\bar{y_{j}}\right)$ is the CDF of $\bar{y_{j}}$; and 0 < β < 0.5 is CDF difference over median of the feature *j* for the positive patients (e.g., β = 30%).

For the robustness and stability evaluation in each machine learning step, and the whole process, the data are divided into two primary partitions, one for the fitting process (determination of the variables and parameters for the best result), called fit partition (Pfit), and the second one separated for the final evaluation of the model, called test partition (Ptest). It is important that test partition is kept apart from the entire process, so that it will reflect how the algorithms will deal with entirely new data. It is also important that partitions do not have the same patient’s data spread on different partitions, to avoid the learning process being contaminated with the information of patients separated from the final test. In other words, the algorithms learn the whole process with patients in the training set who will never be present in the test set.

During the fitting process, the patient’s data in the fit partition are randomly shuffled and sliced into two new partitions, the training partition (Ptrain), which is used for the training of the classifier, and the validation partition (Pval), which serves to measure the classifier prediction performance. The fitting process is repeated 10 times with the shuffled training and validation partition in such a way that the same patient’s data participate in both sides. It is also important to note that all replicates from the same patient are always inside the same partition to avoid data cross-contamination between training and validation, or final test. A low variance between the classification results in the 10 experiments shows that the model trained is stable and generalizes over the fit partition data.

### Data availability.

The data used in this paper can be divided into two sets: mass spectrometry data from PCM patients and healthy volunteers (here referred to as raw data) and the machine-learning-derived data calculated on top of the former. The raw mass spectrometry data from PCM patients and healthy volunteers that support the findings of this study are available upon request of the corresponding authors, A.R.R. and R.R.C. These data are anonymized due to participants’ privacy restrictions and are available free of charge, but due to constraints in the acquisition protocol, the data need to be available only upon request. As the machine-learning-derived data do not involve any sensitive information, they are available directly through the Zenodo open-access repository at https://doi.org/10.5281/zenodo.3763768. The institutional review board (IRB) authorization for the data acquisition was registered under the number CAAE ZIKA 053407/2016 at the University of Campinas, Brazil (58).
